# Bioactivity and Antibacterial Behaviors of Nanostructured Lithium-Doped Hydroxyapatite for Bone Scaffold Application

**DOI:** 10.3390/ijms22179214

**Published:** 2021-08-26

**Authors:** Pardis Keikhosravani, Hossein Maleki-Ghaleh, Amir Kahaie Khosrowshahi, Mahdi Bodaghi, Ziba Dargahi, Majid Kavanlouei, Pooriya Khademi-Azandehi, Ali Fallah, Younes Beygi-Khosrowshahi, M. Hossein Siadati

**Affiliations:** 1 Department of Materials Science and Engineering, K. N. Toosi University of Technology, Tehran P.O. Box 19919-43344, Iran; p.keikhosravani@gmail.com (P.K.); siadati@kntu.ac.ir (M.H.S.); 2 Department of Orthopedics, University Medical Center Utrecht, 3584 CX Utrecht, The Netherlands; 3 Research Center for Pharmaceutical Nanotechnology, Biomedicine Institute, Tabriz University of Medical Sciences, Tabriz 51368, Iran; 4 Department of Chemical Engineering, Sahand University of Technology, Tabriz P.O. Box 51335-1996, Iran; a_kahaie@sut.ac.ir; 5 Tissue Engineering and Stem Cells Research Center, Sahand University of Technology, Tabriz P.O. Box 51335-1996, Iran; 6 Department of Engineering, School of Science and Technology, Nottingham Trent University, Nottingham NG11 8NS, UK; mahdi.bodaghi@ntu.ac.uk; 7 Department of Materials Engineering, University of Tabriz, Tabriz 51368, Iran; z.daargahi95@gmail.com; 8 Materials Engineering Department, Faculty of Engineering, Urmia University, Urmia P.O. Box 57561-51818, Iran; m.kavanlouei@urmia.ac.ir; 9 Research Center for Advanced Materials, Faculty of Materials Engineering, Sahand University of Technology, Tabriz P.O. Box 51335-1996, Iran; p_khademi@hotmail.com; 10 Faculty of Engineering and Natural Sciences, Sabanci University, Istanbul 34956, Turkey; ali.fallah@sabanciuniv.edu; 11 Nanotechnology Research and Application Center (SUNUM), Sabanci University, Istanbul 34956, Turkey; 12 Chemical Engineering Group, Faculty of Engineering, Azarbaijan Shahid Madani University, Tabriz P.O. Box 53751-71379, Iran

**Keywords:** hydroxyapatite, Li doping, mechanical alloying, bioactivity, antibacterial

## Abstract

The material for bone scaffold replacement should be biocompatible and antibacterial to prevent scaffold-associated infection. We biofunctionalized the hydroxyapatite (HA) properties by doping it with lithium (Li). The HA and 4 Li-doped HA (0.5, 1.0, 2.0, 4.0 wt.%) samples were investigated to find the most suitable Li content for both aspects. The synthesized nanoparticles, by the mechanical alloying method, were cold-pressed uniaxially and then sintered for 2 h at 1250 °C. Characterization using field-emission scanning electron microscopy (FE-SEM) revealed particle sizes in the range of 60 to 120 nm. The XRD analysis proved the formation of HA and Li-doped HA nanoparticles with crystal sizes ranging from 59 to 89 nm. The bioactivity of samples was investigated in simulated body fluid (SBF), and the growth of apatite formed on surfaces was evaluated using SEM and EDS. Cellular behavior was estimated by MG63 osteoblast-like cells. The results of apatite growth and cell analysis showed that 1.0 wt.% Li doping was optimal to maximize the bioactivity of HA. Antibacterial characteristics against Escherichia coli (*E. coli*) and Staphylococcus aureus (*S. aureus*) were performed by colony-forming unit (CFU) tests. The results showed that Li in the structure of HA increases its antibacterial properties. HA biofunctionalized by Li doping can be considered a suitable option for the fabrication of bone scaffolds due to its antibacterial and unique bioactivity properties.

## 1. Introduction

Bioceramic scaffolds have been broadly employed for the treatment of hard tissues such as bones, joints, and teeth, owing to their outstanding chemical stability and nontoxicity [1,2]. Among the various bioceramics, hydroxyapatite (HA) with the chemical composition of *Ca*_10_(*PO*_4_)_6_(*OH*)_2_ has attracted a great deal of attention. HA has been recognized as the most stable phase of calcium phosphate under physiological conditions and one of the essential components of hard tissues, including bone and tooth [3,4]. HA characteristics can be enhanced by regulating the size, shape, distribution, and agglomeration of its particles [5,6]. HA nanoparticles are more desirable for biomedical applications because of their larger surface area; they also show superior bioactivity and osteoconductivity [7,8]. Since natural bone possesses a nanocrystalline structure, the preparation of HA at the nanoscale manifests its significant importance. Furthermore, densification and sintering take place better at the nanoscale [9]. Doping with Mg, Sr, and Si effectively improves the mechanical, biological and antibacterial behaviors of the HA nanoparticles [10,11,12]. Due to charge type and ionic radius, HA can accommodate dopants in its structure, leading to changes in biological and antibacterial properties. Interestingly, metal dopants, as bioactive materials, can speed up the formation and proliferation of bone cells [13,14]. Moreover, the bioactive materials enable the formation of layers such as bone apatite on the HA surface resulting in strong interfacial bonding [15,16]. Metal dopants such as Li, Zn, Mn, Si, Mg, and Sr cause enhancement of osteogenesis, the proliferation of osteoblast, and neovascularization. The addition of these dopants to the calcium phosphate structure improves bone healing [17,18]. The Li element is a great candidate for modifying mechanical as well as biological behaviors of HA. Besides, there is a trace amount of Li^+^, as alkali and bioelectric material, in the human body [19,20]. The results obtained in a study conducted by Mayer et al. showed that a low amount of Li does not change the crystal structure of HA [7,21,22] but influences osteoblast cell growth, proliferation, and differentiation via stimulation of the Wnt signaling pathway that causes bone regeneration [18]. Scaffold-associated infection results from bacteria that initially colonize the surface of a scaffold then proceed to form a biofilm. Since bacteria growing in biofilms are tolerant to antibiotics and immune cell clearance, the infections are usually only treated by removing the scaffold in a sophisticated two-step revision surgery which imposes a huge socioeconomic burden [21,22]. Staphylococcus aureus (*S. aureus*), the most common bacterium causing implant-related infection, is resistant to the antibacterial action of methicillin and other related drugs of the penicillin class, and has been recognized as a concern to human health for more than a century [23]. Interestingly, inorganic antibacterial agents (metals and metal oxides) exhibit reduced toxicity, superior chemical stability, and better durability compared to organic ones [24,25]. For instance, Li has potent immune-stimulating capabilities and is highly effective against a wide spectrum of bacteria. More importantly, various studies have shown that Li^+^ can increase the effect of HA on osteoblast proliferation [26].

In this study, HA nanoparticles with different wt.% Li (0.0, 0.5, 1.0, 2.0, 4.0) were synthesized by ball-milling, the five samples being named HA, HA-0.5Li, HA-1Li, HA-2Li, and HA-4Li. The effects of Li on particle size, morphology, bioactivity, cell proliferation, and antibacterial property were studied. Ball-milling was used for nanoscale powder synthesis because of its simplicity and its economical and characteristic aspects. The doping of Li in HA derived from natural resources instead of synthetic HA, as well as the ball-milling technique, are both novel aspects of this work. As HA generated from natural sources can form a strong chemical link with the body’s living bone tissue, the HA material employed in this study was removed from bovine cortical bone via a heat-treating technique [27,28,29,30].

## 2. Results

### 2.1. Characterization

**Figure 1:**
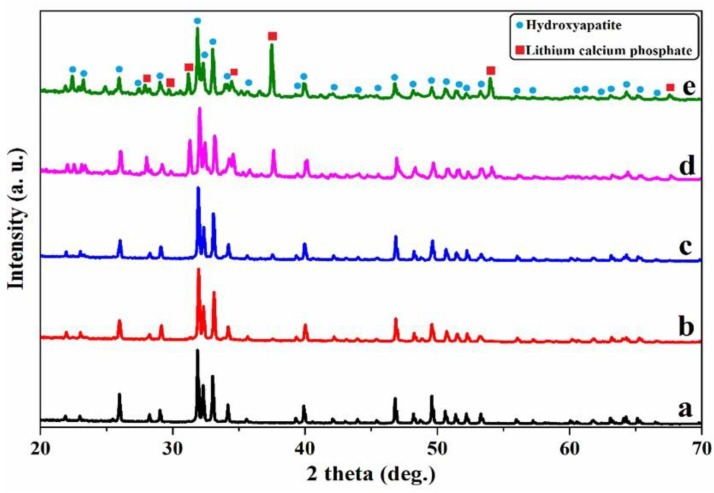
XRD pattern of: (**a**) HA. (**b**) HA-0.5Li. (**c**) HA-1Li. (**d**) HA-2Li. (**e**) HA-4Li sintered at 1250 °C.

The XRD patterns for samples are shown in Figure 1. All the XRD patterns showed the same diffraction planes of HA (*Ca_5_(PO_4_)_3_OH*) with a hexagonal crystal system (JCPDS # 09-0432) and lattice parameters of a = b = 0.9418 nm and c = 0.6884 nm. No new peaks were observed in the HA-0.5Li and HA-1Li samples. However, the two samples HA-2Li and HA-4Li exhibited new peaks belonging to the Li-calcium-phosphate phase (Ca_10_Li(PO_4_)_7_, JCPDS # 045-0550) [5,19]. The lattice volume (*V*) and lattice parameters of the samples are listed in Table 1. Important to note is that adding small amounts of Li dopant changed the lattice parameters of the HA structure. The ionic radius of any dopant smaller than Ca^2+^ (1.14 Å), such as Li^+^ (0.90 Å), leads to the reduction of lattice volume [20,31]. The measured parameters for the HA sample in this study are in accord with those available in the literature [5]; nonetheless, they decreased for the Li-doped samples. The estimated data of the crystallinity percentage (%Xc) of samples are also reported in Table 1. Intense and sharp peaks in the XRD patterns of HA and Li-doped samples corroborated the crystallinity of structures [9]. The presence of Li^+^ also affected the crystallization process of HA. Li-doped samples had a higher crystallinity percentage compared to the HA. Based on the obtained data, the crystallinity increased from 95% to 98% upon adding Li up to 1.0 wt.% but decreased from 98% to 96% for the 4.0 wt.% Li content. The relative density of samples is shown in Table 2. According to this table, the density of HA-0.5Li and HA-1Li samples increased. The density and the Li^+^ radius being smaller than those of Ca^+^, and thus, when up to 1.0 wt.% Li was introduced in the HA structure, the density increased. However, the density decrease with higher Li in the HA structure was attributed to the formation of the Li-calcium-phosphate phase [5,19].

**Figure 2:**
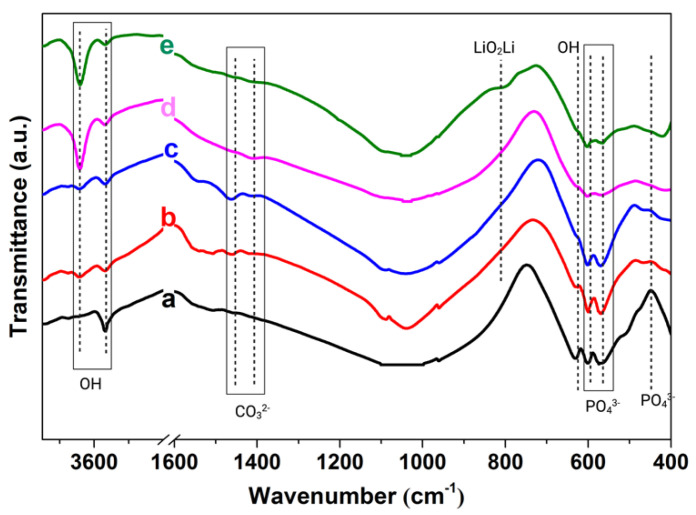
FTIR spectrum of: (**a**) HA. (**b**) HA-0.5Li. (**c**) HA-1Li. (**d**) HA-2Li. (**e**) HA-4Li sintered at 1250 °C.

Figure 2 shows that all five samples had a similar FTIR spectrum. The absorption bands at 440, 573, 602, 961, and 1000–1100 cm^−1^ were assigned to the vibrational modes of tetrahedral phosphate ions (PO_4_^3−^) in HA. The absorption bands characteristic of the liberation and stretching vibration of the hydroxyl group in HA were located at 631, 3567, 3642 cm^−1^. The LiO_2_Li bond was seen at 810 cm^−1^. The absorption band split into two bands at 1462 and 1411 cm^−1^ corresponded to the carbonate (CO_3_^2−^) species originating from atmospheric CO_2_. The mentioned bonds are the main characteristics for the formation of type-B carbonated apatite, in which CO_3_^2-^ substitutes PO_4_^3−^. The bands at 1646, 2980, and 3431 cm^−1^ were for the adsorbed water molecules [8,19,32,33].

**Figure 3:**
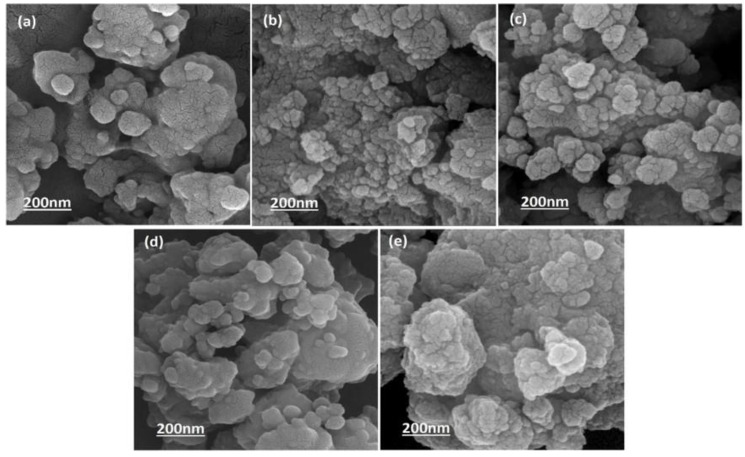
FE-SEM micrograph of: (**a**) HA. (**b**) HA-0.5Li. (**c**) HA-1Li. (**d**) HA-2Li. (**e**) HA-4Li powder before sintering.

**Figure 4:**
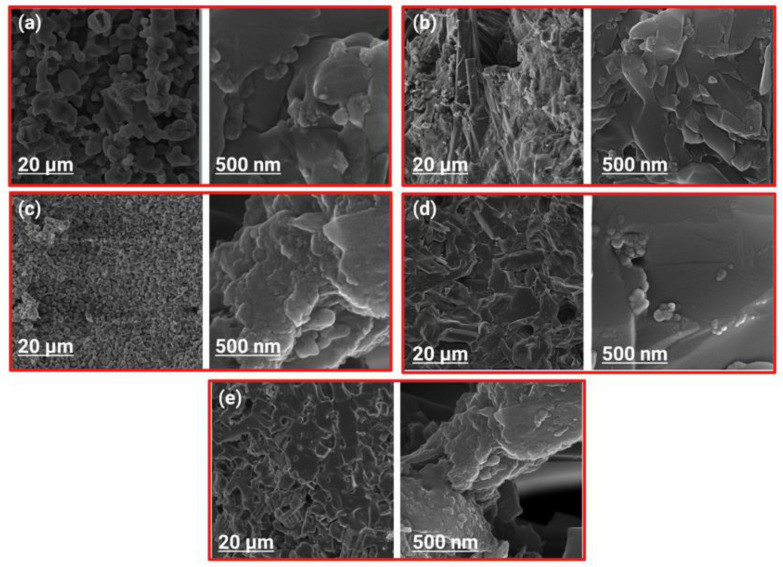
FE-SEM micrograph showing fracture surface of: (**a**) HA. (**b**) HA-0.5Li. (**c**) HA-1Li. (**d**) HA-2Li. (**e**) HA-4Li sample sintered at 1250 °C.

The FE-SEM micrographs in Figure 3 show the surface morphology of the synthesized powders by ball-milling before sintering at 1250 °C. It can be observed that the Li dopant had no considerable effect on the morphology and size of the HA nanoparticles. There are spherical agglomerates on the surface of larger particles possessing various size distributions. Figure 4 shows the FE-SEM micrographs of the fracture surfaces of samples after sintering. The Li-doped samples had heterogeneous microstructures, except for HA-1Li sample. The HA-2Li and HA-4Li samples showed nonuniform grain growth. Particle sizes were between 60 and 120 nm.

### 2.2. Bioactivity Behavior

#### Apatite Growth Ability

**Figure 5:**
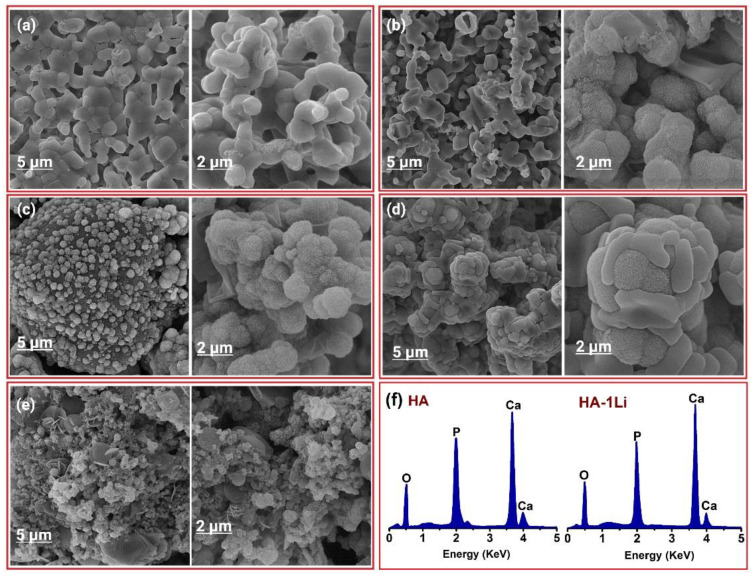
SEM micrograph of: (**a**) HA. (**b**) HA-0.5Li. (**c**) HA-1Li. (**d**) HA-2Li. (**e**) HA-4Li after soaking in SBF for 7 days and (**f**) the EDS of HA and HA-1Li.

Figure 5 shows the SEM micrographs of all samples and EDS for HA and HA-1Li after soaking in SBF for 7 days. The formation of the cauliflower-like apatite layer on the surface, characteristic of bioactivity, was evident. According to Figure 5, the formation of an apatite layer decreased slightly by increasing the Li content from 1.0 to 2.0 and 4.0 wt.%, although the layers were thicker than those of HA and HA-0.5Li samples.

### 2.3. Biological Behavior

#### 2.3.1. Cellular Behavior

**Figure 6:**
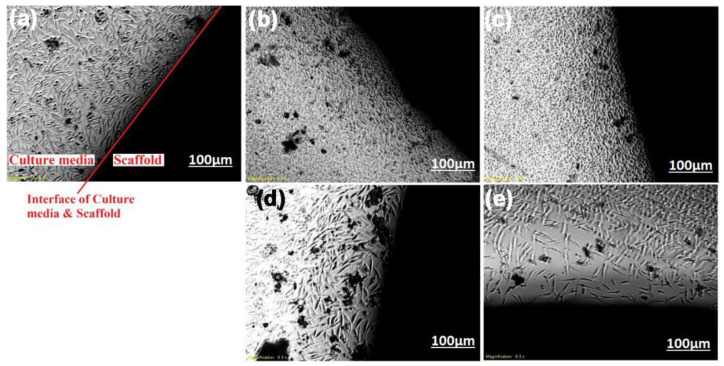
Density of proliferated cells in the interface of: (**a**) HA. (**b**) HA-0.5Li. (**c**) HA-1Li. (**d**) HA-2Li. (**e**) HA-4Li showed by optical microscopy.

To study the growth characteristic of the MG63 osteoblast-like cells (MG63-cells), optical microscopy images were captured from the interface of samples and the culture media. As observed in Figure 6, the HA-0.5Li and HA-1Li samples, compared to the HA sample, indicated suitable cell proliferation. It is known that cell growth on the surface is a significant feature for the investigation of biocompatibility, which influences cell differentiation in the later stages [31]. The highest cell proliferation took place in the HA-1Li sample, but decreased significantly in the HA-2Li and HA-4Li samples.

**Figure 7:**
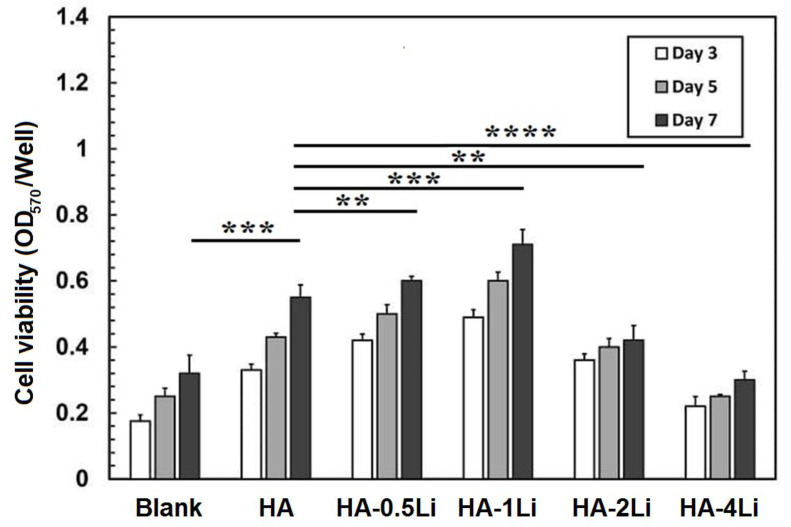
Optical density of cell seeded on: HA; HA-0.5Li; HA-1Li; HA-2Li; HA-4Li. (** for *p* < 0.01; *** for *p* < 0.001 and **** for *p* < 0.0001).

Figure 7 illustrates the optical density of the MG63-cells seeded on samples examined by MTT assay for 3, 5, and 7 days. Cells cultured on every sample increased with increasing culture time. Figure 7 shows that HA-0.5Li and HA-1Li samples had the highest optical density of cell growth. The cell viability of Li-doped samples compared with that of the HA sample at different periods showed that adding 0.5 and 1.0% Li did not lead to any cytotoxic results. Additionally, the optical density, as a criterion for cellular behavior (or cell proliferation), was significantly lower (*p* < 0.05) for the HA-2Li and HA-4Li samples. A *p*-value less than 0.05 (*p* < 0.05) was considered to have statistical significance. The differences were shown by brackets and asterisks in histograms (* for *p* < 0.05; ** for *p* < 0.01; *** for *p* < 0.001 and **** for *p* < 0.0001).

**Figure 8:**
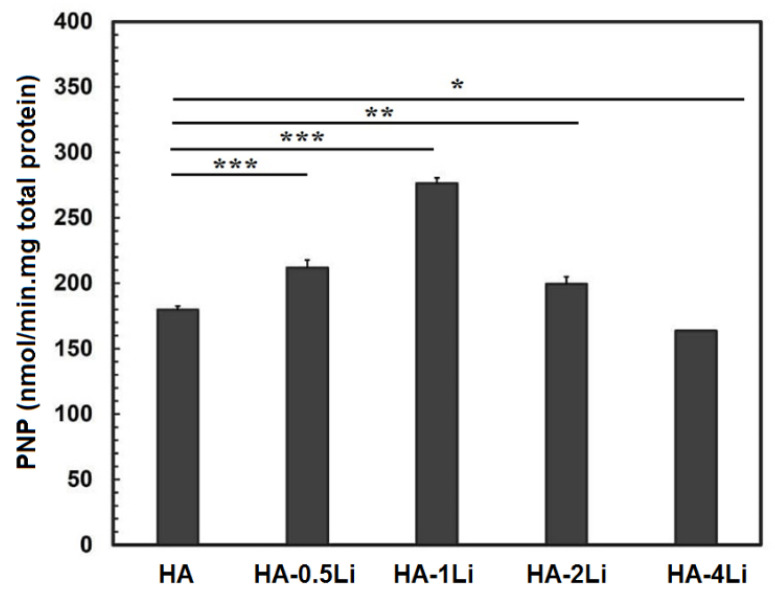
Alkaline phosphatase activity of cells on the HA and HA-Li scaffolds on day 7. (* for *p* < 0.05; ** for *p* < 0.01 and *** for *p* < 0.001).

Figure 8 shows the increase in the alkaline phosphatase (ALP) activity by Li ions, an important marker in bone growth and differentiation. It was confirmed in this study that lower and higher Li compared to its optimal amount (HA-1Li) showed less favorable ALP activity and, therefore, less osteogenesis [18]. Previous studies have shown that in animal models, Li has increased osteogenesis [34]. The results of most studies have shown that the optimal concentration of Li can increase the osteogenesis effects, including osteogenic differentiation, proliferation, and cell protection [35,36]. Li probably affects a wide range of cellular signaling along with the proteins involved, although its exact mechanism of action and concentration dependence are not clear yet [37]. So far, much evidence has been obtained regarding the effect of Li on the Wnt/β-catenin and PI3K-AKT pathways [38,39,40].

#### 2.3.2. Antibacterial Behavior

**Figure 9:**
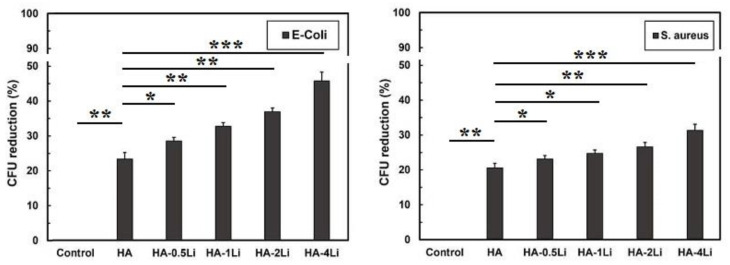
Results of the antibacterial tests for the two *E. coli* and *S. aureus* bacteria. (* for *p* < 0.05; ** for *p* < 0.01 and *** for *p* < 0.001).

Figure 9 shows the results of antibacterial tests against the two *E. coli* and *S. aureus* bacteria. Li doping enhanced the antibacterial properties of HA nanoparticles. Moreover, the *E. coli* bacterium had lower resistance than *S. aureus*.

## 3. Discussion

**Figure 10:**
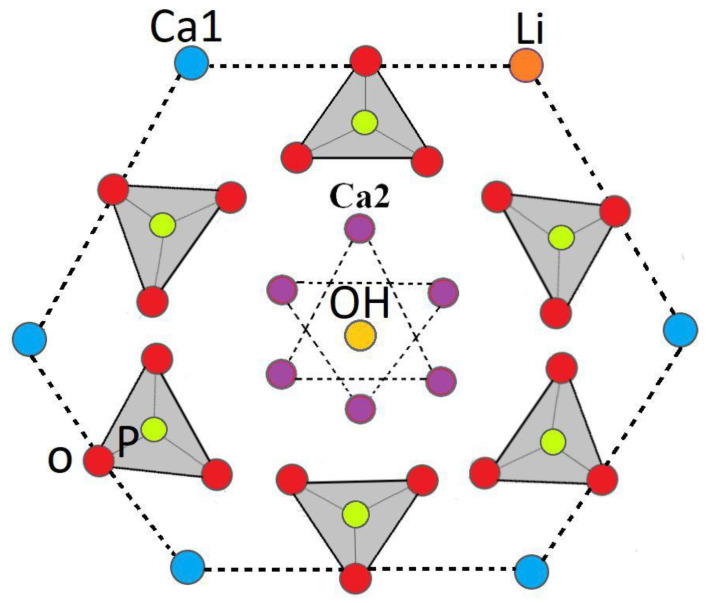
Schematic of Ca1 substitutional sites for Li.

Cation substitution upon doping the HA structure gave rise to changes in the phase composition, biological and antibacterial properties, degree of crystallinity, and grain size. The crystalline structure of HA could be stabilized or destabilized due to the ionic radius of the substituted dopant. The reason for adding any dopants into the HA structure is to improve morphology and densification [41,42]. Based on XRD characterization (Figure 1) and Table 1 and Table 2, HA allows the substitution of dopant in its structure, and thus, its lattice parameters changed. The HA-2Li and HA-4Li samples showed less lattice volume variations. This can be attributed to the formation of Ca_10_Li(PO_4_)_7_ phase, and consequently, the placement of Li^+^ in the HA structure [8,43]. To explain this, it is important to know where the Li ions were placed in the HA structure. The sites depicted in Figure 10 are the locations that can be filled by the dopant ions in the HA structure. Depending on the ionic radius, a dopant can replace Ca2 or Ca1 sites. The cations with a radius larger than the Ca ion would fill Ca2 and the smaller cations in Ca1 sites. As mentioned before, Li^+^ has a smaller ionic radius than Ca^2+^; therefore, it was substituted in the Ca1 places or inserted as an extra atom in the HA structure (interstitial atom) [44,45]. Considering Table 2, due to the formation of another phase (Li-calcium-phosphate) at higher concentrations of Li^+^, the crystallinity of HA decreased because there were not as many Li ions to substitute the Ca ions [7,8]. According to Table 2, the size of HA crystals can be explained as follows. First, the addition of Li (0.5 and 1.0 wt.%) resulted in a more compact structure and smaller crystal size. Second, at the higher Li contents (2.0 and 4.0 wt.%), Li-calcium-phosphate phase formation consumed some of the Li^+^, resulting in lower Li^+^ available for doping in the HA structure. This resulted in lower densification of the HA lattice causing an increase in the HA crystal size [8,43,46]. Moreover, as shown in Figure 3, the grain growth of HA-2Li and HA-4Li samples before sintering was due to the formation of a liquid phase containing Li and Ca phosphates [47]. After sintering (Figure 4), the densification was increased in HA-0.5Li and HA-1Li samples because Li^+^ is small and caused higher diffusion. Considering a study by Lee et al., the higher Li doping provoked more grain growth by diminishing the grain boundary energy [48,49].

Bioactivity is described as the material feature allowing a strong, adherent, and direct bonding with the bone in a living body [18]. Based on the data reported in Figure 5, the formed apatite with an atomic ratio of Ca/P = 1.55 covered some of the HA surface. However, HA-1Li formed a higher amount of apatite. The Ca/P atomic ratio of the apatite deposits in HA-1Li was 1.67, which was the same as the Ca/P atomic ratio of HA in the bone structure. The presence of Ca_10_Li(PO_4_)_7_ and Ca_3_(PO_4_) phases generated in HA-1Li can be attributed to the higher amount of apatite formed in HA-1Li compared to HA. Negative charges were formed on the HA surface to form apatite while immersed in SBF due to phosphate and hydroxyl groups. The negative charges interacted with Ca^2+^, and afterward, the surface acquired positive charges [50]. Due to the displacement of positive charge ions such as Ca^2+^ and Li^+^ released from the surfaces of Li-doped samples with H_3_O^+^ ions, the formation of hydroxyl ions could be stimulated. The hydroxyl ions combine with the positive charge ions in the SBF and form apatite deposits [43]. Furthermore, cellular behavior was investigated by representing the density of proliferated cells as in Figure 6. The numbers of attached cells onto HA-0.5Li and HA-1Li samples confirmed that their surface features were suitable for cellular growth [8,51]. The observations in Figure 6 and Figure 7 can be explained as follows. Low-level doping (HA-0.5Li and HA-1Li) resulted in a change in the surface topography and subsequently increased the HA density and cell proliferation [13,43]. However, high-level doping (HA-2Li and HA-4Li) increased solubility and increased release of Li^+^, causing some of the effects of cellular toxicity in MG63-cells (Figure 7), which led to less proliferation and viability cell. The results showed that HA-1Li was the optimal sample that provided the best biocompatibility and proliferation of cells [43,52,53,54].

**Figure 11:**
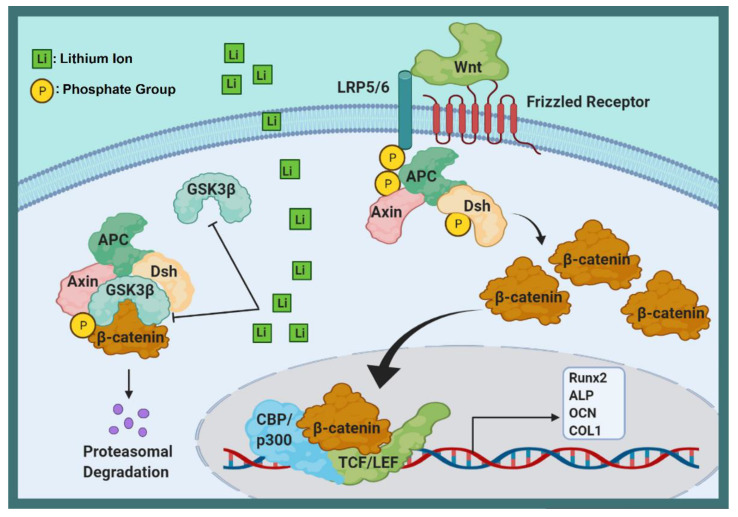
Schematic representation of Li ions interaction with cells.

The schematic in Figure 11 further illustrates the interaction of Li ions with cells. Wnt/beta-catenin is a vital signaling pathway in bone tissue recovery that stimulates proliferation and osteoblastic differentiation [55,56]. Wnt signaling also increases bone differentiation by inhibiting the expression of the PPAR_λ_ adipose-specific gene [57]. Studies have shown that activating the Wnt signaling pathway in adipose-derived mesenchymal stem cells prevents them from differentiating into fat and leads to their differentiation into osteoblast cells [58,59]. Beta-catenin, which plays an essential role in the transcription of genes involved in proliferation and differentiation [60] via a destruction complex, is led by a destruction complex to ubiquitination and finally to proteasomal degradation [61]. This destruction complex is composed of the proteins Axin, protein phosphate 2A (PP2A), adenomatous polyposis coli (APC), glycogen synthase kinase 3 (GSK-3), and casein kinase 1a (CK1a) [62,63]. This destruction complex also causes phosphorylation of beta-catenin, whose degradation by proteasome prevents its accumulation in the cytoplasm [64]. The canonical Wnt signaling pathway activity decomposes the destruction complex and disrupts its function [65]. To activate the Wnt signaling pathway, the Wnt glycoproteins bind to the frizzled receptor and LRP5/6 coreceptors, which phosphorylate the Dishevelled protein [66]. Activated Dishevelled protein inhibits GSK-3B, which prevents the phosphorylation of beta-catenin. As a result, beta-catenin is isolated from APC and accumulates in the cytoplasm [67]. Studies have shown that Li increases accumulated beta-catenin in the cytoplasm by activating Wnt and inhibiting GSK-3B [18,68]. Accumulated beta-catenin eventually enters the nucleus where it binds to transcription factors (TCF-LEFs) for transcription of target genes dependent on the Wnt pathway [69,70]. Studies on MG63-cells have shown that Li ions increase the ALP, OCN, OPN, Col1α1, Runx2, LRP5, and LRP6 proteins. Li also increases mineral nodules and calcium deposition [71,72,73]. However, there are numerous and sometimes contradictory reports about the dual effect of the Wnt pathway on the differentiation of stem cells into bone cells, so that the activation of this pathway first has an increasing role (up to the 14th day) and then a decreasing role on osteogenic differentiation. Li also reduces osteoclastogenesis by affecting the PI3K-AKT pathway [74]. PI3K-AKT is an essential signaling pathway for growth, proliferation, and survival [75]. Li enhances the entire activity of AKT by increasing AKT phosphorylation. Activated AKT inhibits GSK-3β, which subsequently downregulates NFATc1 expression [76,77], and this reduces osteoclast differentiation [78]. In general, it seems that Li, by affecting cellular signaling pathways, causes increasing osteoblast differentiation, proliferation, and cell protection, but decreasing osteoclast differentiation.

**Figure 12:**
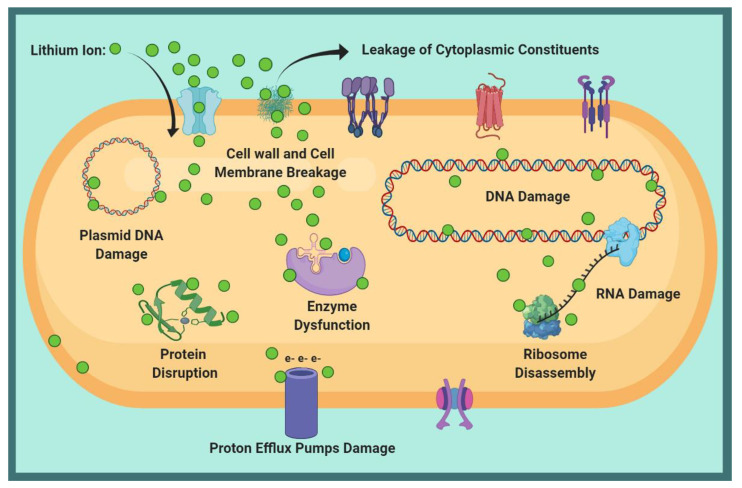
Schematic of the possible Li function against bacteria.

According to the antibacterial results against *E. coli* and *S. aureus* in Figure 9, the bacterial viability decreased by increasing Li ion dopant in the HA structure. Because gram-negative bacteria (*E. coli*) have a thinner peptidoglycan layer than gram-positive bacteria (*S. aureus*), the entry of Li^+^ into *E. coli* and structural damage were less limited [35]. In other words, the purines in the outer layer of gram-negative bacteria acted as a channel for the entry of low molecular weight substances; consequently, the amount of damage to *E. coli* was higher at similar Li contents [79,80,81]. The results of electron microscopy in previous research have shown that bacteria lose their natural shape upon exposure to the metal, related to metal accumulation on the bacterial cell wall [82]. The schematic displayed in Figure 12 shows possible Li function in the face of bacteria. In general, Li destroys the cell wall, leading to leakage of cytoplasmic contents and dehydration [83]. Moreover, the entry of Li ions into bacteria can damage polynucleotide chains such as RNA and DNA. Li disrupts the function of enzymes by altering the conformation of proteins [83,84].

## 4. Materials and Methods

### 4.1. Material Preparation

The HA powder was derived from a natural source, namely a cow’s cortical bone. In a furnace with an air environment, the cortical bone of a cow was burned with an oxyacetylene flame and calcined at 800 °C for 2 h [85]. The other chemicals used in the experiments were all from Merck, Darmstadt, Germany: Li carbonate (*Li_2_CO_3_*) and stearic acid (*C_18_H_36_O_2_*). To make samples with 0.0, 0.5, 1.0, 2.0 and 4.0 wt.% Li, five mixtures containing measured amounts of micro powder HA, Li carbonate, and 2 wt.% stearic acid were prepared. According to previous studies, stearic acid was used as the process control agent (PCA) to prevent agglomeration of the powder [27]. The HA and HA with Li carbonate were milled using a high-energy planetary ball mill (Fritsch Pulverisette, Ider Oberstein, Germany, inert tungsten carbide cups and balls) with a ball to powder ratio of 10:1, under argon gas for 20 h at 300 rpm. One cup contained HA powder, while the other included HA and Li carbonate particles. To prepare samples from each group of synthesized powder, 0.6 g was pressed at 150 MPa to form tablets 15 mm in diameter. Afterward, the tablets were sintered for 2 h at 1250 °C.

### 4.2. Characterization

The phase composition, crystallite size, unit cell volume, lattice strain, and crystallinity percentage of samples were determined by X-ray diffraction (PANalytical Company, Netherlands) using CuKα (*λ* = 1.542 Å). The lattice constants (a, b, and c) of samples were measured by standard HCP unit-cell plane spacing relationship using Equation (1) [86]:

1/*d*^2^ = (4(*h*^2^ + *hk* + *k*^2^))/(3 *a*^2^) + (*l*^2^/*c*^2^) (1)

where *d* is lattice spacing and *h*, *k*, and *l* are Miller indices of the diffractions. The calculated values are listed in Table 1. The unit cell volume (*V*) was determined by Equation (2):

(2) $$V=0.866\cdot a^{2}c$$

The average crystal size (*D*) was calculated using the Scherrer equation, Equation (3):

*D* = 0.94 *λ*/(*β* cos(*θ*)) (3)

in which *λ* is the wavelength of *CuKα* radiation (*λ* = 1.542 Å), *θ* is Bragg diffraction angle, and *β* is full width at half maximum (FWHM) as in Equation (4):

(4) $$\beta^{2}=\beta_{exp}^{2}-\beta_{standard}^{2}\;$$

where *β_exp_* and *β_standard_* are the measured and the instrumental widths, respectively [87]. The crystallinity percentage (%*X_c_*) was estimated using Equation (5) [88]:

%*X_c_* = (*k*/*β*_002_)^3^ (5)

where k is a constant (=0.24), and *β*_002_ is the FWHM of the (002) plane. The density (*ρ*) was measured using the Archimedes method as in Equation (6) [51]:

*Density*(*g*/*cm*^3^) = (*wt*.(*air*)/(*wt*.(*air*) − *wt*.(*water*))*ρ*(*water*)) (6)

The *wt*.(*water*) and *wt*.(*air*) are weights of the samples in water and air, respectively. *ρ*(*water*) is the density of water. The theoretical density of all samples was considered as 3.156 g/cm^3^. Fourier transfer infrared spectroscopy (FTIR, Perkin-Elmer Spectrum 400, Waltham, MA, USA) was used for determining the functional group of the samples over the wavenumber range of 400–4000 cm^−1^. Field-emission scanning electron microscopy (FE-SEM, Tescan, Brno, Czech Republic) with an accelerating voltage of 15 kV was employed to analyze the microstructure and morphology of samples.

### 4.3. Bioactivity Behavior

#### Apatite Growth Ability

To evaluate apatite growth ability, samples were immersed in 20 mL SBF prepared according to the extensively used recipe by Kokubo [89]. The ionic concentrations in SBF were almost like human blood plasma. The specimens were stored in an incubator for 7 days at 37 °C. During the incubation period, the SBF was refreshed every day. After the 7th day, they were washed in deionized water (DI) and dried at room temperature. Afterward, the samples were analyzed by scanning electron microscopy (SEM, Tescan, Brno, Czech Republic) and EDS to observe the formation and growth of bioactive apatite.

### 4.4. Biological Behavior

#### 4.4.1. Cellular Behavior

MG63-cells were used to evaluate the effects of HA and Li-doped HA samples in culture medium on cell proliferation. Before immersing in the culture media, all samples were autoclaved for 15 min at 121 °C. MG63-cells were seeded in a 24-well plate (Falcon, Franklin Lakes, NJ, USA) with a density of 2 × 105 cells/well, containing Dulbecco’s modified eagle media (DMEM) with 10% fetal bovine serum and 1% Pen-Strep antibiotic. Optical microscopy (model-1X832-Deck Inverted Microscope, Olympus, Hamburg, Germany) monitored the cell developments in culture sets. Afterward, the plates were stored in a humidified incubator (n-biotek NB-203XL) for 7 days at 37 ± 1 °C and 5% CO_2_. The cell aggregation in the interface of culture media/specimens was watched at the end of day 7. The cellular behavior of all samples was examined by colorimetric MTT assay (3-(4,5 dimethylthiazol-2-yl)-2, 5-diphenyl tetrazolium bromide) (Sigma Inc., Marlborough, MA, United States) using MG63-cells in the third, fifth, and seventh day of culture. After incubation, the culture medium was exchanged with 100 µL of conditioned culture medium containing 10% MTT solution and kept for 4 h. Then, the culture medium was eliminated, and 100 µL of dimethyl sulfoxide (DMSO) was added to dissolve the formazan crystals. An ELISA microplate reader (Sunrise-Tecan, Austria) calculated the optical absorbance of the resulting blue-violet solution at a wavelength of 570 nm. All samples were also exposed to MG63-cells for 7 and 14 days to assess the ALP activity. After incubation, the MG63-cells were washed with PBS, lysed by lysis buffer, incubated for 30 min at 37 °C and held for 12 h at 4 °C. Afterward, the cells were settled into a tube and centrifuged at 12,000 rpm for 10 min. Then, the supernatant and p-nitrophenyl phosphate solution (1 to 20) were mixed and incubated for 1 h at 25 °C. The ALP activity was determined utilizing the fluorescence microscope system (Cytation 5, BioTek, Winooski, VT, USA) at a wavelength of 405 nm.

#### 4.4.2. Antibacterial Behavior

On HA and Li-doped HA samples, the antibacterial characteristics against *E. coli* and *S. aureus* were performed based on direct interaction of nanoparticles with bacteria by the colony-forming unit (CFU) test [90]. For this purpose, the nanoparticles produced by the ball milling process were first washed with ethanol and DI water, and then dried. A suspension of nanoparticles with a concentration of 100 µg/mL in DI water was prepared, and 10^6^ CFU/mL of bacteria were added to it, followed by incubation for 6 h at 150 rpm shaking speed. A suspension containing only bacteria was considered as the control sample. Eventually, 100 µL suspension of each sample was spread onto LB plates and allowed to grow for 12 h at 37 °C. Finally, colonies were counted and compared with those on the control sample to determine the CFU reduction percentage.

The ALP, MTT, and antibacterial results were expressed as the mean of three independent experiments and plotted as mean ± standard deviation (SD). One-way analysis of variance (ANOVA) was performed using Minitab 17 software. Student’s t-test was used for comparison between experimental groups and control groups. A *p*-value less than 0.05 (*p* < 0.05) was considered to have statistical significance. The differences were shown by brackets and asterisks in histograms (* for *p* < 0.05; ** for *p* < 0.01; *** for *p* < 0.001 and **** for *p* < 0.0001).

## 5. Conclusions

A ball milling technique followed by sintering at 1250 °C was applied to prepare HA and Li-doped HA samples. The samples were characterized by XRD, FE-SEM and FTIR. The results indicated that the crystallinity achieved was over 95% and the crystallites were in the range of 59–89 nm. The 1.0 wt.% Li-doped sample proved the optimal sample because it showed the smallest crystallite size and the highest crystallinity (over 98%). In addition, in vitro bioactivity tests conducted in SBF, MTT, and the ALP assay demonstrated the highest bioactivity and cell proliferation for the optimal sample. Results of the antibacterial assay also showed that Li doping enhanced the antibacterial properties of HA. The antibacterial effect on gram-negative *E. coli* was more than on the gram-positive *S. aureus*. Consequently, the 1.0 wt.% Li-doped HA might be a favorable material for bone scaffold application due to its optimal biological properties.

## Figures and Tables

**Table 1:** Diffraction characteristics of HA and Li-doped HA samples.

Sample	Lattice Parameters (nm)	Unit Cell Volume (nm)	Crystallinity (%)
HA	a = b = 0.9422; c = 0.6886	0.5298	95
HA-0.5Li	a = b = 0.9415; c = 0.6882	0.5287	97
HA-1Li	a = b = 0.9411; c = 0.6880	0.5281	98
HA-2Li	a = b = 0.9417; c = 0.6883	0.5290	96
HA-4Li	a = b = 0.9420; c = 0.6887	0.5297	96

**Table 2:** The calculated values of average crystallite size (D) and relative density (*ρ*) of HA and Li-doped HA samples.

Sample	Crystallite Size (nm)	Relative Density (%)
HA	88.5	90.12
HA-0.5Li	71.2	92.32
HA-1Li	59.3	95.48
HA-2Li	75.6	91.17
HA-4Li	81.4	90.87

## Data Availability

All data generated or analyzed during this study are included in the present article.
